# Changes in Antioxidative Compounds and Enzymes in Small-Leaved Linden (*Tilia cordata* Mill.) in Response to Mistletoe (*Viscum album* L.) Infestation

**DOI:** 10.3390/plants10091871

**Published:** 2021-09-10

**Authors:** Liubov Skrypnik, Pavel Maslennikov, Pavel Feduraev, Artem Pungin, Nikolay Belov

**Affiliations:** Institute of Living Systems, Immanuel Kant Baltic Federal University, Universitetskaya Str., 2, 236040 Kaliningrad, Russia; PMaslennikov@kantiana.ru (P.M.); PFeduraev@kantiana.ru (P.F.); APungin@kantiana.ru (A.P.); NBelov@kantiana.ru (N.B.)

**Keywords:** hemiparasite, small-leaved lime, urban ecology, haustoria, oxidative stress, water deficiency, Foyer–Halliwell–Asada cycle

## Abstract

Mistletoe infestation leads to a decrease in the growth of woody plants, their longevity, and partial or complete drying of the top, as well as premature death. Various environmental stress factors, both abiotic and biotic, stimulate the formation of reactive oxygen species and the development of oxidative stress in plant tissues. This study aimed to investigate the effect of mistletoe (*Viscum album* L.) infestation on the response of the antioxidative defense system in leaves of small-leaved linden (*Tilia cordata* Mill.). Leaves from infested trees were taken from branches (i) without mistletoe, (ii) with 1–2 mistletoe bushes (low degree of infestation), and (iii) with 5–7 mistletoe bushes (high degree of infestation). The relative water content and the chlorophyll *a* and *b* contents in leaves from linden branches affected by mistletoe were significantly lower than those in leaves from non-infested trees and from host-tree branches with no mistletoe. At the same time, leaves from branches with low and high degrees of infestation had significantly higher electrolyte leakage, malondialdehyde and hydrogen peroxide content, oxidized forms of ascorbic acid (dehydroascorbic and 2,3-diketogulonic acids), and oxidized glutathione. The results of principal component analysis show that the development of oxidative stress was accompanied by an increase in proline content and in superoxide dismutase, ascorbate peroxidase, glutathione peroxidase, and glutathione reductase activity. Several biochemical parameters (proline, ascorbic acid, dehydroascorbic acid, glutathione, glutathione peroxidase, ascorbate peroxidase, and dehydroascorbate reductase) were found to be altered in leaves from host-tree branches with no mistletoe. This result indicates that the mistletoe infestation of trees not only causes local changes in the locations of hemiparasite attachment, but also affects the redox metabolism in leaves from other parts of the infested tree.

## 1. Introduction

European mistletoe (*Viscum album* L.) is a spherical hemiparasitic evergreen bush that develops stable haustoria in the host tree. Mistletoe is currently widely distributed in Europe, Asia, and America. The geographic distribution of mistletoe is limited primarily by temperature. The low average temperatures of the coldest and warmest months of the year limit the distribution of European mistletoe [1]. Consequently, the warmer the climate is, the wider the range mistletoe will have, increasing the area of infested trees [1].

Mistletoe infestation causes a decrease in the growth of trees and their longevity, and partial or complete drying of the treetop, as well as premature death [2,3]. Mistletoe is believed to capture water, mineral nutrients, and carbohydrates (glucose, fructose, sucrose) from their host trees, thereby disrupting the osmotic and carbon balance of the host [4,5]. Urban trees are especially infested by mistletoe, as the conditions of their growth place them in a vulnerable position [6]. Limited water and nutrients, insufficient space for roots and crown, and air pollution weaken urban trees and make them more susceptible to biotic factors, enhancing the negative consequences of mistletoe infestation [6,7].

Previous studies [8] showed that in the territory of Kaliningrad, the trees of the small-leaved linden (*Tilia cordata* Mill.) are more susceptible to mistletoe infestation compared to other urban trees. The natural ranges of the small-leaved linden are located in the forests of the temperate zone of Europe and adjacent regions of Asia [9,10]. In addition, the small-leaved linden is an important species for urban and recreational forestry and open landscapes. These trees are often planted singly or in groups along roads and in urban parks [11]. Linden trees are distinguished by several advantages, particularly a compact leaf crown, shade tolerance, tolerance to various soil conditions, and resistance to wind and stressful urban conditions, such as smoke, dirt, dust, and air pollution [12]. Despite this, the soft wood, relatively old age, and inadequate crown care of the trees lead to the spread of mistletoe.

Plant resistance to pathogens is controlled by the innate immune system, with two levels of defense. The first level is associated with inducing pattern-triggered immunity and the second with inducing effector-triggered immunity [13]. In the last decade, the elements of these systems have been established in some parasitic plant–host plant interactions [13,14]. It is known that products of cell wall decomposition formed under the action of hydrolases of parasitic plants can participate in damage-associated molecular patterns (DAMPs) and induce the defense mechanism of the host plant [15]. Although the genes encoding cell wall-destructing enzymes are expressed in mistletoe haustoria [16], the mechanisms of signal perception and transduction through DAMP for mistletoe–host plant interactions have not been established. After haustoria develops in the host plant tissues, several defense mechanisms in the host plant can be activated. In particular, the release of cytotoxic compounds, the formation of mechanical barriers through lignification, the production of reactive oxygen species (ROS), and the initiation of a hypersensitive response can be observed [14].

A standard characteristic of various environmental stress factors, both abiotic and biotic, is their ability to stimulate the ROS formation in plant tissues [17]. On the one hand, the production and accumulation of ROS in plants leads to serious destruction of cell organelles and causes membrane peroxidation, which leads to damage to the cell membrane, degradation of biological macromolecules, and ultimately cell death [18,19]. On the other hand, ROS act as signal molecules that activate stress-dependent metabolic pathways and cellular defense mechanisms [19]. The mechanisms of plant resistance under stress conditions include several physiological and biochemical strategies, and many enzymatic components, such as superoxide dismutase (SOD, EC 1.15.1.1), catalase (CAT, EC 1.11.1.6), peroxidase (POD, EC 1.11.1.7), glutathione peroxidase (GPX, EC 1.11.1.9), glutathione reductase (GR, EC 1.6.4.2), glutathione S-transferase (GST, EC 2.5.1.18), ascorbate peroxidase (APX, EC 1.11.1.11), monodehydroascorbate reductase (MDAR, EC 1.6.5.4), and dehydroascorbate reductase (DHAR, EC 1.8.5.1), as well as non-enzymatic components such as ascorbic acid, glutathione, phenolic compounds, carotenoids, α-tocopherols, and free amino acids, including proline [18]. Proline, as an osmoprotectant, plays a crucial role in the protection of plant cells under stress conditions that cause an osmotic imbalance in cells (drought, salinity, cold, high temperature, etc.) [20]. It has been also considered to be a non-enzymatic antioxidant due to its capacity to scavenge ROS (singlet oxygen, hydroxyl radical) and reduce the effect of ROS by stabilizing the antioxidative enzymes and protecting the integrity of cell membranes [21,22].

Some studies have demonstrated that the infestation of trees by mistletoe of different genera (*Viscum album, Phoradendron perrottetii*) leads to changes in the oxidative status, the level of some low-molecular-weight antioxidants, and the activity of enzymatic antioxidants in host trees [3,23,24,25,26]. It was found that in the case of infestation of Scots pine (*Pinus sylvestris* L.) with European mistletoe, the levels of hydrogen peroxide and glutathione increased in the bark of trees, and the level of the reduced form of ascorbic acid decreased [25]. One study [26] showed an increase in hydrogen peroxide and malondialdehyde content in the bark of small-leaved linden, Norway maple (*Acer platanoides* L.), and black poplar (*Populus nigra* L.) trees severely infested with European mistletoe. Fir needles also showed elevated oxidative stress parameters (electrolyte leakage, malondialdehyde, superoxide anion radical, hydrogen peroxide, and hydroxyl radical) and the activity of some antioxidative enzymes (CAT, POD) [3]. When *Tapirira guianensis* trees were infested by *P. perrottetii* mistletoe, a higher content of phenolic compounds (tannins and flavonoids) was observed in the branches of host trees [23].

To the best of our knowledge, there have been no previous studies on the effects of mistletoe infestation on small-leaved linden trees in terms of the development of oxidative stress and the response of the antioxidative system in the leaves of host trees. This study aimed to investigate the effect of mistletoe infestation on (i) the relative water and chlorophyll content in linden leaves; (ii) the parameters of oxidative stress (electrolyte leakage, malondialdehyde, hydrogen peroxide); (iii) the contents of non-enzymatic antioxidants (proline, reduced and oxidized forms of glutathione, three forms of ascorbic acid, total phenolic compounds); and (iv) the activity of antioxidative enzymes (SOD, CAT, POD, as well as enzymes of the ascorbate–glutathione cycle). The results of this study will expand our knowledge about the mechanisms of the response of host trees to mistletoe infestation and help to develop strategies for adapting to it.

## 2. Results

### 2.1. Effect of Mistletoe Infestation on the Relative Water Content and the Content of Chlorophylls in Linden Leaves

Relative water content in leaves from linden branches infested by mistletoe was significantly lower compared to control plants and leaves from branches with no mistletoe (Table 1).

The contents of chlorophyll *a* and chlorophyll *b* were significantly reduced under mistletoe infestation (Table 1), and the rate of decrease of chlorophyll *a* depended on the intensity of infestation. The content of chlorophyll *b* in the leaves of mistletoe-infested branches was lower compared to the control variants but did not differ significantly in the experimental variants with different degrees of infestation (Table 1). With a high degree of mistletoe infestation, a decrease in the chlorophyll *a*/*b* ratio was also observed (Table 1), associated with a decrease in the total amount of chlorophyll and a more pronounced decrease in chlorophyll *a* compared to chlorophyll *b* (Table 1).

### 2.2. Effect of Mistletoe Infestation on Oxidative Stress Parameters in Linden Leaves

Mistletoe infestation of trees resulted in elevated electrolyte leakage compared to control groups; the larger the infestation, the higher the level of electrolyte leakage (Table 2).

Similar changes were found for the content of malondialdehyde, a product of lipid peroxidation, and hydrogen peroxide; these components in leaves from branches infested by mistletoe showed significantly higher content compared to control variants (C and NI) (Table 2). The levels of hydrogen peroxide and malondialdehyde were 1.4 and 2 times higher, respectively, in the presence of heavy mistletoe infestation than in the leaves of non-infested trees (Table 2). This indicates that during mistletoe infestation, along with hydrogen peroxide, other peroxide compounds that oxidize the lipophilic components of cell membranes are generated in leaves.

### 2.3. Effect of Mistletoe Infestation on Non-Enzymatic Antioxidants in Linden Leaves

In comparison with non-infested trees, significantly higher proline content was found in the leaves collected from branches on which mistletoe was present (LI and HI) and host-tree branches with no mistletoe (NI) (Table 3).

The content of reduced glutathione significantly decreased when the tree was infested with mistletoe; the greater the intensity of the infestation, the lower the content in leaves of the studied trees (Table 3). The content of oxidized glutathione was significantly higher in linden leaves from branches with mistletoe infestation compared to control variants (Table 3).

This work aimed at studying the content of three forms of ascorbic acid in the leaves of linden trees infested by mistletoe, particularly the reduced form of ascorbic acid, dehydroascorbic acid, and 2,3-diketogulonic acid. The content of the reduced form of ascorbic acid was 1.5–2 times higher in infested trees than in the leaves of non-infested trees (Table 3). The maximum content of ascorbic acid was determined in leaves from the control branches of host-trees under a low degree of mistletoe infestation. With greater infestation, the level of ascorbic acid decreased slightly (Table 3). The content of dehydroascorbic acid was also higher in the leaves of infested trees, including leaves collected from branches with no mistletoe bushes (Table 3); its maximum level was observed at low and high degrees of infestation (LI). The maximum content of 2,3-diketogulonic acid was observed in leaves with severe mistletoe infestation (HI) (Table 3).

The highest total content of phenolic compounds was found in leaves harvested from linden branches with a low degree of mistletoe infestation (LI) (Table 3). With a high degree of infestation (HI), the content of phenolic compounds sharply decreased and was significantly lower compared to the control variants (C and NI).

### 2.4. Effect of Mistletoe Infestation on Antioxidative Enzyme Activity in Linden Leaves 

The response of antioxidative enzymes to mistletoe infestation was assessed through the activity of SOD, CAT, enzymes involved in the Foyer–Halliwell–Asada cycle, and POD (Table 4). SOD activity was significantly higher in linden leaves from branches with mistletoe infestation (LI and HI) compared to control variants (C and NI) (Table 4). No significant differences were found in variants with different degrees of infestation.

CAT activity was also significantly higher in leaves from branches infested with mistletoe (Table 4); by comparison, at high infestation intensity, there was a decrease in CAT activity.

The Foyer–Halliwell–Asada cycle includes the enzymes APX, MDAR, DHAR, GPX, and GR. A similar reaction of enzymes to mistletoe infestation was observed for those directly involved in the utilization of hydrogen peroxide, specifically APX and GPX (Table 4). Thus, APX and GPX activity was significantly higher in the case of mistletoe infestation, both in leaves from branches with no mistletoe (NI) and with low and high degrees of infestation (LI and HI) (Table 4). Maximum MDAR activity was observed in linden leaves from branches with low infestation (Table 4). High levels of DHAR activity were found in linden leaves from branches with a low degree of infestation (LI), as well as leaves from branches with no mistletoe but the tree was infested (NI) (Table 4). Significantly higher GR activity was observed only in leaves from branches with a high degree of infestation (Table 4). In the leaves of other variants, no significant differences in GR activity were found. 

The activity of POD, the substrates of which are phenolic compounds and hydrogen peroxide, increased sharply in the leaves from branches with a low degree of mistletoe infestation (approximately 2.5 times compared to non-infested trees) (Table 4).

### 2.5. Interaction between Relative Water Content, Chlorophyll Content, Oxidative Stress, and Antioxidative Response of Linden Trees to Mistletoe Infestation

**Figure 1:**
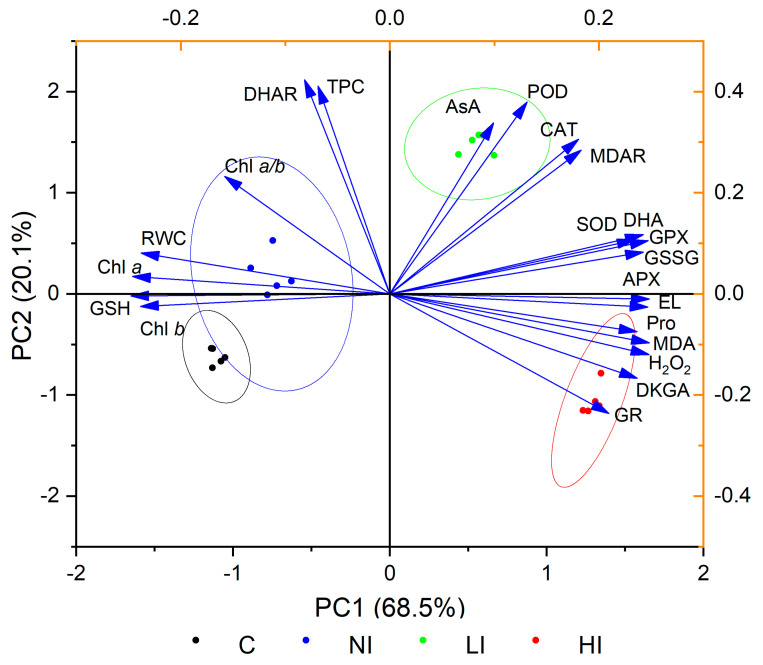
Principal component analysis (PCA) biplot of studied parameters grouped by variants of experiment. C, control, non-infested trees; NI, leaves from non-infested branches of infested trees; LI, leaves from branches with 1–2 mistletoe bushes (low degree of infestation); HI, leaves from branches with 5–7 mistletoe bushes (high degree of infestation). RWC, relative water content; Chl *a*, chlorophyll *a*; Chl *b*, chlorophyll *b*; Chl *a*/*b*, chlorophyll *a*/*b* ratio; EL, electrolyte leakage; MDA, malondialdehyde; Pro, proline; GSH, glutathione; GSSG, oxidized glutathione; AsA, ascorbic acid; DHA, dehydroascorbic acid; DKGA, 2,3-diketogulonic acid; TPC, total phenolic compounds; SOD, superoxide dismutase; CAT, catalase; APX, ascorbate peroxidase; MDAR, monodehydroascorbate reductase; DHAR, dehydroascorbate reductase; GPX, glutathione peroxidase; GR, glutathione reductase; POD, peroxidase.

A multivariate statistical analysis (principal component analysis (PCA)) was constructed to study the interactions among variables in terms of the response to the mistletoe infestation of linden trees. The results of PCA are presented as a two-dimensional biplot (Figure 1). About 97.9% of the total variance can be explained by the first six principal components. Among all principal components, the first one (PC1) contributed 68.5% and the second (PC2) contributed 20.1% of the variance. The 95% bootstrapped confidence intervals were 62.2–74.3% and 14.9–27.8% for PC1 and PC2, respectively (*n* = 999). The loadings on the biplot indicated that oxidative stress parameters (electrolyte leakage, malondialdehyde, hydrogen peroxide), oxidized forms of ascorbic acid and glutathione, and some antioxidants (proline, SOD, GPX, APX, GR) were positively correlated with each other and negatively with relative water, chlorophyll, and glutathione content. The score plot represents the authenticated grouping of various variants. The only exceptions were the control groups (C and NI), with some overlap of 95% confidence ellipses.

## 3. Discussion

European mistletoe is a hemiparasitic plant capable of photosynthesis but completely dependent on the water and mineral nutrients of the host tree. One of the most important consequences of mistletoe infestation of trees is the occurrence of water scarcity, which is especially exacerbated in arid regions and/or dry years [3,27]. In this study, it was shown that the relative water content in the leaves of small-leaved linden infested by mistletoe was lower than in leaves from non-infested trees (Table 1). This result is consistent with previous data obtained in the study of Scots pine infested with mistletoe (*V. album* subsp. *Austriacum*) [3,25]. The increased water deficiency in the host tree is also related to the fact that, as shown earlier, the stomata of mistletoe leaves remain open in an almost unregulated manner and maintain a high transpiration rate under various environmental conditions [28]. As a rule, water deficit leads to decreased chlorophyll content in the leaves due to their destruction [29]. In this work, it was shown that the levels of chlorophyll *a* and *b* were lower in leaves of trees infested by mistletoe (Table 1). In addition, a positive correlation was found between relative water content and the level of pigments (Figure 1 and Supplementary Table S1). This result also indirectly confirms the development of stress in infested trees due to water imbalance. However, for more accurate conclusions, it is necessary to conduct additional research aimed at studying physiological parameters associated with water exchange such as the water potential of leaves, transpiration, and efficient use of water.

It is now well known that the main plant defense responses to biotic stress are the rapid production of ROS (ROS-burst) and an increase in the level of the stress-related phytohormone ethylene and the contents of secondary metabolites (callose, phytoalexins, lignins, etc.), as well as the activation of marker genes and signaling pathways controlled by the phytohormones salicylic acid (SA) and jasmonic acid (JA). As a result, all of these processes lead to the initiation of a hypersensitive response (HR) and systemic acquired resistance (SAR) to biotic stress factors [30]. However, there is relatively little research on the mechanisms of plant defense against mistletoe, especially when compared to studies on such mechanisms against herbivores and pathogens. The results of this research show that the leaves of mistletoe-infested trees have elevated levels of hydrogen peroxide, malondialdehyde, a lipid peroxidation product, and electrolyte leakage compared to non-infested trees (Table 2). However, it should be noted that an increase in ROS production and the development of oxidative stress are not specific responses to biotic stress but are also observed when plants are exposed to various abiotic factors, including drought or mineral nutrient deficiency [31,32]. In this regard, it is difficult to conclude whether the detected increase in ROS was a direct response to the mistletoe attack or the consequences associated with its development on the host tree.

The regulation of plant redox homeostasis under stress is based on the activation of the antioxidative system, which includes several non-enzymatic compounds and antioxidative enzymes. Proline is a low-molecular-weight cyclic amino acid that is known to provide osmotic regulation in plants under the influence of stress factors (drought, salinity, cold, high temperature, etc.). It reduces the effects of ROS by stabilizing the antioxidative enzymes and protecting the integrity of cell membranes [20,21,22]. In the present study, proline levels were higher in the leaves of infested trees, including leaves collected from the branches of infested trees with no mistletoe bushes (Table 3). It is understood that data on the change in the proline content in the leaves of host trees infested with mistletoe have not been previously published. However, considering the hypothesis about the development of water stress in such trees, the increase in the proline level established in this study becomes quite understandable.

Protection against ROS and mediation of the activation of defense genes are the main functions of glutathione for plants under stress. Stressful situations often cause oxidative stress, which changes the glutathione content and the glutathione ratio toward the oxidized form [33]. A decrease in the level of the reduced form of glutathione and an increase in its oxidized form, in the case of mistletoe infestation of trees, indicate a shift of redox homeostasis in the cell toward oxidation processes (Table 3). A somewhat different relationship was found for various forms of ascorbic acid; the content of the reduced form was higher in leaves of infested trees than non-infested ones (Table 3). However, the level of oxidized forms (dehydroascorbic and 2,3-diketogulonic acids) and the level of oxidized glutathione increased when trees were infested by mistletoe (Table 3). This result may indicate an intensification of the ascorbic acid biosynthesis process in trees infested by mistletoe, despite a decrease in the water and chlorophyll content. Such an increase in biosynthesis is possible due to the action of jasmonates, which participate in regulating the biosynthesis of ascorbic acid and are important signal molecules in plants under biotic stress [34]. Notably, in [25], it was revealed that hydrogen peroxide accumulation in the bark of mistletoe-infested Scots pine was accompanied by increased reduced glutathione content and decreased reduced ascorbic acid content. The opposite result obtained in this work can be associated with both the specific feature of the reaction of small-leaved linden to infestation and the fact that different plant materials (leaves and bark) were used in the studies.

Phenolic secondary metabolites are an integral part of the plant’s defense system against pathogens, including bacteria, fungi, and viruses, as well as against major abiotic stresses such as drought, salinity, and UV radiation [35]. In this study, the total content of phenolic compounds was higher in leaves from branches with a low degree of mistletoe infestation and lower in leaves from branches with five to seven mistletoe bushes (high degree of infestation) compared to the control (Table 3). It was shown in [23,36] that the infestation of *T. guianensis* trees by the mistletoe *P. perrottetii* caused no significant changes in the level of phenolic compounds in the leaves of the host trees but led to an increase in the level of tannins in non-infested host branches. Changes in the content of phenolic compounds in host trees may be associated with various processes in which these secondary metabolites are involved, particularly, maintenance of the redox status in the cell, lignification, signaling, and the initiation of systemic acquired resistance [37]. An increase in the content of phenolic compounds in leaves from branches with a low degree of mistletoe infestation could be associated with activation of their biosynthesis through the activation of phenylalanine ammonia-lyase (PAL), which is a key enzyme in the phenylpropanoid pathway. Previously, it was shown that PAL activity increases under mild drought stress and decreases under severe drought stress [38]. In addition to a decrease in PAL activity at high stress levels, the decrease in the content of phenolic compounds under a high degree of mistletoe infestation could be due to other reasons. First, there are not enough reducing agents in cells for the regeneration of phenolic compounds oxidized by ROS. Second, the oxidized forms of some phenolic compounds (flavonoids and hydroxycinnamic acids) can be involved in the biosynthesis of polymeric compounds, in particular lignin or suberin [39], which is characteristic of plant responses to biotic stress.

As a rule, the metabolites considered above (ascorbic acid, glutathione, phenolic compounds) not only directly exhibit antioxidative properties, but also act as substrates for antioxidative enzymes. This study revealed an elevation in SOD, CAT, APX, MDAR, GPX, POD, and GR activity in the leaves of trees infested by mistletoe (Table 4). The activity of the latter enzyme increased only with heavy infestation of the branches. At the same time, the APX and GPX activity was also higher in leaves collected from the branches of host trees on which there were no mistletoe bushes. In a previous study on changes in the activities of some enzymes (POD and SOD) in leaves of various tree species infested by mistletoe, it was shown that the greatest changes were peculiar to SOD [24]. Other authors noted an increase in CAT and POD activity in the needles of infested Scots pine trees [3]. The increase in activity of enzymes that catalyze the primary stages of ROS detoxification, particularly superoxide anion radical and hydrogen peroxide, revealed in the present study indicates a successful response of the antioxidative system to oxidative stress caused by mistletoe infestation. However, an increase in the contents of oxidized forms of glutathione and ascorbic acid indicates insufficiently effective work of the enzymes responsible for the regeneration of the reduced forms of these compounds. The pattern of changes in the activity of different antioxidative enzymes was differed according to the intensity of infestation. SOD, APX, GPX, and GR activity was higher with a high degree of infestation compared to a low degree. APX is known to have a higher affinity with hydrogen peroxide than POD and CAT and may play a more important role in regulating redox homeostasis in plant cells under strong stress conditions [40]. In addition to scavenger hydrogen peroxide, GPX is also able to detoxify lipid peroxides, which is important under high stress levels [41]. A decrease in the activity of other studied enzymes (CAT, MDAR, DHAR, and POD) under a high degree of mistletoe infestation may be associated with the inactivation of these enzymes by ROS, decreased enzyme synthesis, or an insufficient amount of substrates (for example, reduced forms of glutathione and phenolic compounds) [41,42].

The results of PCA showed that the development of oxidative stress was followed by an increase in proline content and SOD, GPX, APX, and GR activity (Figure 1). Variants of the experiment (C, NI, LI, HI) were divided into groups indicating the different changes in the studied biochemical parameters under different degrees of mistletoe infestation. The overlap of the 95% confidence ellipses between the two control groups (C and NI) indicates that some parameters of leaves from host-tree branches with no mistletoe bushes remained at the same level as that in non-infested trees. However, it should be noted that some biochemical changes also occurred in leaves from the control branches of host trees. In particular, they had higher proline, ascorbic acid, and dehydroascorbic acid content, lower reduced glutathione content, and higher APX, GPX, and DHAR activity compared to non-infested trees. This result indicates that the infestation of trees by mistletoe not only leads to local changes in the places of hemiparasite attachment, but also affects the redox metabolism in the leaves from other parts of infested tree.

## 4. Materials and Methods

### 4.1. Plant Material

Leaves from small-leaved linden trees (*Tilia cordata* Mill.) were used as the object of the study. This tree species was selected as it is more susceptible to European mistletoe infestation (*Viscum album* L. subsp. *Album*) in Kaliningrad [8]. The trees under study were located on a test plot of approximately 250 m × 250 m, situated in the territory of Kaliningrad (54°44′56″ N, 20°30′55″ E). For the study, 5 control trees with no mistletoe (in the text, tables and figure, this variant is designated as C) and 5 trees infested by mistletoe were selected. For sampling, control and infested linden trees of approximately the same age (60–70 years) growing at a distance of no more than 50 m from each other were selected. Leaves from infested trees were taken from branches on which (i) there was no mistletoe (NI), (ii) there were 1–2 mistletoe bushes (low degree of infestation, LI), and (iii) there were 5–7 mistletoe bushes (high degree of infestation, HI). On each investigated tree, one branch was selected that matched the required criteria (number of mistletoe bushes, direction to sunlight, approximately the same age). From each branch, 8–12 leaves were collected. The leaves from each branch were combined, mixed, and used for further analysis as an average sample. Sampling of leaf material was conducted at the beginning of June. Part of the leaves intended for biochemical analysis was placed in liquid nitrogen for 5–10 min after collection and stored in the laboratory refrigerator at −80 °C until further analysis.

### 4.2. Determination of Relative Water Content

Part of the freshly harvested leaves was used to establish the relative water content. First, the fresh mass of leaves was measured. Then, the leaves were kept in distilled water for 12 h. After that, the samples were dried with filter paper and weighed to determine the turgid weight. The dry weight of leaves was measured after drying at 70 °C for 24 h. Relative water content in leaves was calculated using the formula given in [43].

### 4.3. Determination of Chlorophyll Content

The chlorophyll *a* and *b* content in the extract of fresh leaves preserved at −80 °C after homogenization with 80% acetone and centrifugation was determined spectrophotometrically. Optical absorption was measured at 470, 646.8, and 663.2 nm. The chlorophyll content was calculated using the formula in [44] and expressed per gram of dry weight.

### 4.4. Determination of Oxidative Stress Parameters

Electrolyte leakage was determined in freshly harvested leaves by measuring electrical conductivity as described in [45]. Determination of the malondialdehyde content in fresh leaves preserved at −80 °C was carried out by reaction with thiobarbituric acid as described in [46] with an extinction coefficient of 155 mM^−1^ cm^−1^. The hydrogen peroxide content was determined using potassium iodide (KI) solution according to [47]. The malondialdehyde and hydrogen peroxide content was expressed per gram of dry weight.

### 4.5. Determination of Non-Enzymatic Antioxidant Content 

The proline content in fresh leaves preserved at −80 °C was determined spectrophotometrically using the acid-ninhydrin method as described in [48]. The calculation of proline content was carried out according to a calibration graph constructed using standard solutions of L-proline. 

The determination of reduced and oxidized glutathione content was carried out spectrophotometrically according to [49]. The reduced glutathione content was determined by using sequential reactions of oxidation by 5,5’-dithio-bis (2-nitrobenzoic acid) and enzymatic reduction by NADPH in the presence of GR. The reduced glutathione content was calculated as the difference between the total glutathione and oxidized glutathione content.

The ascorbic, dehydroascorbic, and 2,3-diketogulonic acid content was measured spectrophotometrically by the reaction of dehydroascorbic and 2,3-diketogulonic acids with 2,4-dinitrophenylhydrazine as described in [39]. Ascorbic acid was first oxidized with 2,6-dichlorophenolindophenol. For the separate determination of dehydroascorbic and 2,3-diketogulonic acids, the former was reduced to ascorbic acid using a solution of unithiol in phosphate buffer.

The total content of phenolic compounds was determined using Folin–Ciocalteu reagent as described in [50]. The phenolic compound content was calculated from a calibration curve constructed using gallic acid as a standard.

The content of all non-enzymatic antioxidants was recalculated on a dry weight basis for a better comparison of data regarding their content in plant samples differing in moisture.

### 4.6. Determination of Antioxidative Enzyme Activity

Frozen leaf samples (approximately 0.4 g) were ground in liquid nitrogen and homogenized in 10.0 mL of ice-cold 100 mM phosphate buffer (pH 7.0) containing 0.1 mM EDTA and 1.0% polyvinylpyrrolidone [51]. To prevent the inactivation of enzymes when determining APX, MDAR, and DHAR activity, 1 mM ascorbic acid and 2 mM β-mercaptoethanol were added to the homogenizing buffer [52]. The resulting homogenates were centrifuged at 15,000× *g* for 20 min at 4 °C. Supernatants were used to determine antioxidative enzyme activity and protein concentration.

SOD (EC 1.15.1.1) activity was determined by its ability to inhibit photochemical reduction of nitro blue tetrazolium according to a previously described protocol [53]. CAT (EC 1.11.1.6) activity was determined by decreased optical absorption at 240 nm as a result of the decomposition of hydrogen peroxide and was calculated using an extinction coefficient of 39.4 mM^–1^ cm^–1^ [54]. APX (EC 1.11.1.11) activity was measured by decreased optical absorption at 290 nm as a result of oxidation of the ascorbate with hydrogen peroxide and calculated by using an extinction coefficient of 2.8 mM^−1^ cm^−1^. MDAR (EC 1.6.5.4), and GR activity was determined by decreased NADPH concentration and calculated using an extinction coefficient of 6.2 mM^−1^ cm^−1^ (at 340 nm). The activity of DHAR (EC 1.8.5.1) was assessed by the increase in optical absorption at 265 nm as a result of the formation of ascorbate (extinction coefficient equal to 14.6 mM^−1^ cm^−1^). APX, MDAR, DHAR, and GR activity was measured according to the protocols described in [52]. GPX (1.11.1.9) activity was assessed by the decreased reduced glutathione content as a result of its oxidation with hydrogen peroxide [55]. POD (EC 1.11.1.7) activity was determined spectrophotometrically using guaiacol and hydrogen peroxide as substrates and was calculated using an extinction coefficient of 26.6 mM^−1^ cm^−1^ (at 470 nm) [56]. 

To convert the activity of all enzymes per mg of protein, the protein concentration was measured spectrophotometrically using the Bradford assay [57]. Bovine serum albumin was used as a standard.

A Shimadzu UV-3600 spectrophotometer (Shimadzu, Kyoto, Japan) was used for spectrophotometric analysis. All biochemical analyses were performed in triplicate.

### 4.7. Statistical Analysis

The experimental data were statistically processed using OriginPro 9 software (OriginLab Corporation, Northampton, MA, USA). Statistical analysis of data was performed only with biological replications corresponding to the number of studied control and infested trees (*n* = 5). The tables show mean values with standard error of the mean. To determine the significance of differences between mean values, one-way ANOVA followed by Tukey’s post hoc test was carried out. Principal component analysis (PCA) was used to assess the relationships between the studied parameters. The significance of loadings in PCA was estimated using the bootstrap at *n* = 999 and presented as 95% confidence intervals [58]. The bootstrap analysis was performed using Past v. 4.01 (Natural History Museum, University of Oslo, Oslo, Norway).

## Figures and Tables

**Table 1:** Effect of mistletoe infestation on relative water content and chlorophyll content in linden leaves.

Treatment	RWC, %	Chlorophyll *a*, mg g^−1^ DW	Chlorophyll *b*, mg g^−1^ DW	Total Chlorophyll, mg g^−1^ DW	Chlorophyll *a/b* Ratio
C	89.2 ± 0.7 a	2.25 ± 0.03 a	1.56 ± 0.02 a	3.80 ± 0.04 a	1.44 ± 0.01 a
NI	89.1 ± 1.0 a	2.52 ± 0.04 a	1.51 ± 0.05 a	3.75 ± 0.05 a	1.51 ± 0.03 a
LI	83.6 ± 0.5 b	1.79 ± 0.06 b	1.23 ± 0.03 b	3.03 ± 0.07 b	1.45 ± 0.02 a
HI	77.8 ± 0.9 c	1.52 ± 0.05 c	1.14 ± 0.03 b	2.66 ± 0.06 c	1.33 ± 0.03 b

RWC, relative water content; DW, dry weight; C, control, non-infested trees; NI, leaves from non-infested branches of infested trees; LI, leaves from branches with 1–2 mistletoe bushes (low degree of infestation); HI, leaves from branches with 5–7 mistletoe bushes (high degree of infestation). Different letters in each column indicate significant differences between variants based on Tukey’s post hoc test (*n* = 5) at *p* ≤ 0.05.

**Table 2:** Effect of mistletoe infestation on oxidative stress parameters in linden leaves.

Treatment	Electrolyte Leakage, %	MDA, nmol g^−1^ DW	H_2_O_2_, nmol g^−1^ DW
C	12.4 ± 0.7 c	93.6 ± 2.0 c	2.24 ± 0.03 a
NI	15.8 ± 0.6 c	99.4 ± 3.1 c	2.25 ± 0.03 a
LI	23.8 ± 1.3 b	137.8 ± 4.4 b	1.79 ± 0.05 b
HI	30.8 ± 1.4 a	183.8 ± 3.6 a	1.52 ± 0.6 c

MDA, malondialdehyde; DW, dry weight; C, control, non-infested trees; NI, leaves from non-infested branches of infested trees; LI, leaves from branches with 1–2 mistletoe bushes (low degree of infestation); HI, leaves from branches with 5–7 mistletoe bushes (high degree of infestation). Different letters in each column indicate significant differences between variants based on Tukey’s post hoc test (*n* = 5) at *p* ≤ 0.05.

**Table 3:** Effect of mistletoe infestation on contents of non-enzymatic antioxidants in linden leaves.

Treatment	Proline, µmol g^−1^ DW	GSH, µmol g^−1^ DW	GSSG, µmol g^−1^ DW	AsA, µg g^−1^ DW	DHA, µg g^−1^ DW	DKGA, µg g^−1^ DW	TPC, mg g^−1^ DW
C	155.2 ± 5.1 c	53.8 ± 1.6 a	4.10 ± 0.11 b	111.6 ± 3.0 c	44.2 ± 1.4 c	36.8 ± 2.0 c	9.48 ± 0.39 b
NI	219.0 ± 5.5 b	45.6 ± 1.2 b	4.12 ± 0.14 b	218.6 ± 5.8 a	63.8 ± 2.7 b	38.4 ± 1.5 c	9.20 ± 0.41 b
LI	239.8 ±5.4 b	32.8 ± 1.4 c	5.70 ± 0.10 a	230.6 ± 3.7 a	79.4 ± 1.5 a	61.4 ± 2.3 b	12.92 ± 0.86 a
HI	321.4 ± 4.7 a	23.0 ± 1.5 d	5.84 ± 0.12 a	178.2 ± 5.2 b	84.6 ± 1.5 a	110.1 ± 4.2 a	5.44 ± 0.29 c

DW, dry weight; GSH, reduced glutathione; GSSG, oxidized glutathione; AsA, ascorbic acid; DHA, dehydroascorbic acid; DKGA, 2,3-diketogulonic acid; TPC, total phenolic compounds. C, control, non-infested trees; NI, leaves from non-infested branches of infested trees; LI, leaves from branches with 1–2 mistletoe bushes (low degree of infestation); HI, leaves from branches with 5–7 mistletoe bushes (high degree of infestation). Different letters in each column indicate significant differences between variants based on Tukey’s post hoc test (*n* = 5) at *p* ≤ 0.05.

**Table 4:** Effect of mistletoe infestation on activities of antioxidative enzymes in linden leaves.

Treatment	SOD, U mg^−1^ Protein	CAT, nmol H_2_O_2_ mg^−1^ Protein min^−1^	APX, µmol AsA mg^−1^ Protein min^−1^	MDAR, µmol NADPH mg^−1^ Protein min^−1^	DHAR, µmol AsA mg^−1^ Protein min^−1^	GPX, µmol GSH mg^−1^ Protein min^−1^	GR, µmol NADPH mg^−1^ Protein min^−1^	POD, µmol Guaiacol mg^−1^ Protein min^−1^
C	4.46 ± 0.05 c	131.1 ± 2.9 c	14.1 ± 1.0 d	3.47 ± 0.06 c	1.96 ± 0.03 b	1.33 ± 0.03 d	2.22 ± 0.04 b	0.58 ± 0.04 c
NI	4.44 ± 0.04 c	134.2 ± 2.8 c	24.1 ± 1.3 c	3.49 ± 0.04 c	2.77 ± 0.06 a	2.18 ± 0.03 c	2.21 ± 0.03 b	0.68 ± 0.01 bc
LI	7.57 ± 0.06 a	227.8 ± 3.9 a	36.6 ± 1.4 b	4.50 ± 0.05 a	2.87 ± 0.04 a	3.30 ± 0.05 b	2.29 ± 0.02 b	1.48 ± 0.05 a
HI	7.46 ± 0.11 b	174.0 ± 2.7 b	48.5 ± 1.8 a	3.97 ± 0.04 b	1.49 ± 0.05 c	3.53 ±0.04 a	2.68 ± 0.04 a	0.80 ± 0.02 b

SOD, superoxide dismutase; CAT, catalase; APX, ascorbate peroxidase; MDAR, monodehydroascorbate reductase; DHAR, dehydroascorbate reductase; GPX, glutathione peroxidase; GR, glutathione reductase; POD, peroxidase. C, control, non-infested trees; NI, leaves from non-infested branches of infested trees; LI, leaves from branches with 1–2 mistletoe bushes (low degree of infestation); HI, leaves from branches with 5–7 mistletoe bushes (high degree of infestation). Different letters in each column indicate significant differences between variants based on Tukey’s post hoc test (*n* = 5) at *p* ≤ 0.05.

## Data Availability

The data presented in this study are available on request from the corresponding author.

## Supplementary Materials

The following are available online at https://www.mdpi.com/article/10.3390/plants10091871/s1, Table S1: Correlation matrix with Pearson coefficient values for relative water content, chlorophyll content, oxidative stress, and antioxidative compounds and enzymes.
